# Empowering the voiceless. Disorders of consciousness, neuroimaging and supported decision-making

**DOI:** 10.3389/fpsyt.2022.923488

**Published:** 2022-09-06

**Authors:** Timo Istace

**Affiliations:** ^1^Department of Law, Research Group Personal Rights and Property Rights, University of Antwerp, Antwerp, Belgium; ^2^Antwerp Health Law and Ethics Chair (AHLEC), Antwerp, Belgium

**Keywords:** informed consent, disorders of consciousness, neuroimaging, competence, supported decision-making

## Abstract

Patients suffering from (Prolonged) Disorder of Consciousness are deemed incompetent to give valid informed consent due to the presumed impairment of their cognitive functions and the impossibility to communicate with them. Neuroscientists have, however, discovered ways in which communication with some of these patients might be possible by using neuroimaging. This would for the first time make it possible to include them in the decision-making on their own medical treatment. In this article, I elaborate on the prospect of communicating with patients with impaired consciousness in order to obtain their informed consent. I first map the current state-of-the-art in neuroimaging research that exhibits the possibility of communicating with some of these patients. Secondly, I examine how obtaining informed consent from these patients might be possible, given that the specificities and limitations of communication *via* neuroimaging render the task of assessing their competence rather difficult. Thirdly, I identify some of the important ethical and legal considerations that have to be taken into account before introducing neuroimaging in clinical practice as a means to obtain informed consent. Lastly, I look into the concept of supported decision-making and how this concept relates to the use of neurotechnology to support minimally conscious patients in their abilities to decide over their own medical treatment.

## Introduction

The treatment trajectory for patients suffering from Disorders of Consciousness (DoC) faces a variety of hurdles. Consequently, this group of especially vulnerable people “suffer from high rates of misdiagnosis, inadequate medical surveillance, undertreatment of pain, inadequate rehabilitation, and segregation in chronic care” [(1), p. 1732]. In this article, I will focus on another, particularly difficult barrier in the treatment of patients suffering from Prolonged Disorder of Consciousness (i.e., DoC lasting for at least 28 days)^1^ as a result of an acquired brain injury, namely obtaining informed consent for medical treatment. As obtaining consent directly from these patients is impossible due to the impossibility to communicate with them, and the subsequent uncertainty about their cognitive abilities, medical professionals resort to substitute decision-making by legal representatives. Since this constitutes a major interference with these patients' potential autonomy, this course of action is not to be taken lightly. This is especially the case since substitute decision-making as the default in patients suffering from PDoC might come under pressure in the wake of neuroimaging research that demonstrates the possibility of directly communicating with patients with impaired consciousness. Since this communication might generate reliable and consistent answers from the patients concerning their wishes and preferences, the prospect emerges of including them in the decision-making on their own medical treatment.

In this contribution, I will discuss the consequences of introducing neuroimaging in clinical practice as a means of obtaining informed consent from patients suffering from PDoC. Acknowledging that this technology and accompanying decoding methods are in their infancy, it does nonetheless seem highly plausible that communication *via* neuroimaging will one day be introduced in the treatment of patients suffering from PDoC (2). The following analysis will examine the ethical and legal issues that need to be taken into account before this technology may be considered for use in clinical practice. Firstly, I will elaborate on the role of neuroimaging in diagnosing PDoC and establishing lines of communication with patients suffering from PDoC. Secondly, after briefly looking into some general principles governing informed consent, I will discuss the informed consent procedures in this group of patients, as well as the ethical and legal concerns and considerations that need to be addressed before the introduction of neuroimaging in these informed consent procedures in medical practice. Lastly, I will scrutinize the concept of supported decision-making in the light of these developments.

## Disorders of consciousness and neuroimaging

Three states of DoC can be distinguished, i.e., coma, Vegetative State (VS) – also referred to as Unresponsive Wakefulness Syndrome –, and Minimally Conscious State (MCS) (3)^2^. Especially the last two states of impaired consciousness are of significant importance in the following analysis. Both patients in VS and patients in MCS namely exhibit signs of wakefulness. This is accounted for by, for instance, sleep-wake cycles and occasional reflex movements. MCS is to be distinguished from VS on the basis of preservation of conscious awareness. While patients in VS do not show any form of self or environmental awareness, patients in a MCS produce behavioral responses which occur inconsistently, but can nonetheless be reproduced or sustained long enough, and are of such a degree of complexity so that they cannot be considered reflexive behaviors. For example, patients suffering from MCS can possess the ability to follow simple demands (e.g., command to move a finger or to blink an eye), to engage in intelligible verbalisation, or to generate purposeful behavior that is meaningful in relation to environmental stimuli (e.g., reaching for objects or smiling as a reaction to linguistic or visual stimuli) (5). In comatose patients, on the other hand, there is no manifestation of wakefulness, nor of awareness. These different states of PDoC find themselves on a continuum (6), which can make it challenging to distinguish between VS and MCS (1). In fact, the misdiagnosis in patients with VS is relatively high, as some research shows that up to 40% of patients diagnosed as vegetative do in fact show signs of minimal consciousness (7)^3^.

Ground-breaking neuroimaging research conducted in 2006 by Owen and colleagues using fMRI brought a whole new dimension to the diagnosis of PDoC. Their research was among the first to uncover the presence of covert consciousness in a patient who was considered to be in a VS and thus assumed to lack any mental activity on the basis of common behavioral assessment (8). However, mental imagery generated by fMRI revealed that this patient was capable of wilfully modulating her mental activity as a response to commands, in a way that resembles healthy subjects. Monti and colleagues in their study found that 10% of the patients that appeared to be vegetative showed signs of awareness and were able to conduct mental imagery tasks in an fMRI scanner (9). Later, it was argued that 14–17% of patients suffering from PDoC showed brain activity that might account for consciousness when participating in these studies (10, 11). Another study demonstrated that also EEG could be used to uncover awareness in patients suffering from VS (which is of significant interest considering the practical and financial advantages of EEG as compared to fMRI) (12). Building on this approach, Monti and colleagues succeeded in engaging in wilful communication with a patient with covert consciousness, on the basis of yes-or-no questions (9, 13). Comparing their brain activity patterns with those observed in healthy subjects who had been asked similar questions, enables neuroscientists to decode answers to yes-or-no questions in minimally conscious patients, and thus to establish a basal, unilateral line of communication. These patients, “who show non-behavioural evidence of consciousness or communication only measurable *via* para-clinical testing (i.e., functional MRI, positron emission tomography, EEG or evoked potentials) can be considered to be in a functional locked-in syndrome” [(14), p. 1373]. The notion “functional locked-in syndrome”^4^ is introduced to indicate patients with extreme motor dysfunction in whom preserved higher brain functions are identified by functional neuroimaging techniques (16). It is thus a category of patients that has to be distinguished from patients suffering from locked-in syndrome. Although parallels exist and the treatment of locked-in patients might be interesting to draw upon when it comes to addressing ethical and legal questions that may arise in the context of DoC, locked-in syndrome is strictly speaking not categorized as a DoC since it does not entail an impairment of consciousness.

This ground-breaking research that enables direct communication with a small^5^–but significant–group of minimally conscious patients, holds tremendous prospects for people suffering from impaired cognitive capacities that prevent them from communicating *via* behavioral ways with their environment. It gives rise to the possibility that one day, reliable and consistent BCI communication with these patients may be feasible^6^,^7^. In any case, as Peterson and colleagues point out, already today, neuroimaging allows us for instance to ask whether they are in pain (18). This is an accomplishment of major importance as this could greatly improve the pain management and, consequently, the well-being of a patient. Moreover, in the aftermath of the first research findings suggesting that communication with some patients with minimal (covert) consciousness was possible, scholars already started speculating about the possibility of re-including those patients in medical decision-making (1, 19, 20). This is a complicated issue as it gives rise to an important question: Is it, taking all the specificities and limitations of neurotechnology-enabled communication into account, ethically permissible and legally conceivable to deploy neuroimaging to obtain informed consent from patients with impaired consciousness? This question is generally met with considerable skepticism. In the next part, the obstacles for obtaining valid informed consent from patients suffering from PDoC *via* neuroimaging tools will be discussed. I will examine whether these barriers are truly of such a magnitude that we ought to be very skeptical about the aspiration of using neurotechnological tools to include patients suffering from PDoC in their own medical decision-making, or whether these obstacles can be surmounted in the foreseeable future so that neurotechnology might empower the voiceless within their medical decision-making process.

It has to be stressed that the neuroimaging techniques and corresponding decoding techniques referred to above are currently not applied within clinical practice as the empirical and conceptual robustness of these techniques is not yet satisfactory. Moreover, this might not be the case for some time as the technology and decoding methods require further development. Therefore, I want to acknowledge that this analysis works under the–uncertain, but highly plausible–assumption that, in the future, functional neuroimaging techniques may be used in clinical practice to communicate with patients with minimal (covert) consciousness. In this light, it is important to proactively reflect on the ethical and legal issues that arise. As the optimisation of patients' autonomy and well-being–core values within health care–ought to be the ultimate goal, neuroimaging should be operationalised in informed consent procedures when proven technically possible. However, this has to be done in a responsible way.

## Competence and informed consent

The obligation of medical professionals to seek informed consent of patients before a medical treatment or a clinical research trial, is a cornerstone of medical ethics and health law (21). It is rooted in the principle of respect for the patient (22). This principle prescribes acknowledging people's autonomy while at the same time protecting those persons whose autonomy is diminished (23). The principle of informed consent has globally been embraced as a core principle within bioethics and is enshrined in various supranational legal instruments (24)^8^.

Commonly, the legal capacity required for valid informed consent is assessed within a competence-based framework (25). Within this framework, medical professionals must, before performing a medical treatment, obtain informed consent from patients who are competent. Patients are legally competent to provide informed consent when they have the decision-making capacity required to consider them autonomous decision-makers. Patients with significantly impaired decision-making capacity are deemed incompetent (26). Whereas, competence is a legal condition for valid informed consent, decision-making capacity is a medical concept that needs to be appreciated from a medical point of view. The assessment of the legal notion of competence thus relies on the clinical appreciation of decision-making capacity. In general, the law operates under a rebuttable presumption that adults possess decision-making capacity. It is only when there are signs that suggest that the patient's decision-making capacity is impaired, that medical professionals have to make an in-depth assessment (18). As decision-making capacity is considered task-specific, this capacity assessment must be tailored to the specific decision that is to be made. In the context of persons with–potentially–impaired decision-making capacity, medical decisions require a different threshold for decision-making capacity due to the fact that medical decisions are of a different level of complexity (25).

Decision-making capacity is generally assessed on the basis of the ability to understand the relevant information, to appreciate the consequences of the treatment options, to reason about these options, and to communicate a choice (21). These four elements are considered the underlying psychological abilities required for adequate decision-making capacity^9^. When patients do not meet the competence threshold for a certain medical procedure on the basis of these criteria, medical professionals must seek informed authorization from a substitute decision-maker who bases their judgement on the previously expressed wishes and preferences of the patient or, if no wishes had been expressed, the best interests of the patient (27).

Various tools exist to evaluate a patient's decision-making capacity. Depending on the context, different assessment tools may be used. Although there is not one unique accepted standardized instrument (28), the *MacArthur Competence Assessment Tool for Treatment* (MacCAT-T), a tool created by Grisso and Applebaum (29), stands out as the golden standard for the assessment of patients' competence within the clinical context (18). Using the MacCAT-T, medical professionals evaluate a patient's decision-making capacity by conducting a semi-structured interview that aims at establishing whether the patient is able to *understand, reason, appreciate* the given information and treatment options, and *communicate* their choice. If the test shows the patient's level of decision-making capacity does not reach the threshold required by the decision that ought to be made, the patient is deemed incompetent to consent and the medical professional turns to the substitute decision-maker to seek informed authorization.

## Disorders of consciousness and informed consent

Today, patients suffering from VS or MCS are presumed to lack decision-making capacity (30). The absence of behavioral responses that allow medical professionals to verbally engage with the patients, and that might account for the consistent mental activity and cognitive functions required for decision-making capacity, eliminates the possibility of obtaining informed consent, e.g., by means of the MacCAT-T prescribed interview. Consequently, a medical professional will need to rely on substitute decision-making when it concerns patients suffering from PDoC. However, this *presumption of lack of decision-making capacity* in patients suffering from PDoC is no longer self-evident following ground-breaking research that reveals that neuroimaging enables the identification of covert consciousness in some patients who appear to be vegetative, and makes communication with some of these minimally conscious patients possible. In this light, it can no longer categorically be ruled out that patients with impaired consciousness possess the cognitive capabilities required to give informed consent.

In this regard, it is important to stress that practical hurdles remain as the methods for *reading* the patients' answers from their brain activity are far from being established. For instance, the fatiguability of patients suffering from PDoC makes that even basal communication on the basis of yes-or-no questions remains particularly challenging (31). Anticipating that these practical and technological hurdles will be overcome considering the rapid developments within neurotechnology, using this technology to obtain informed consent will still remain a major challenge. Obtaining informed consent namely requires more than just establishing a line of communication. The main challenge is to examine whether the presence of the required psychological and cognitive capacities can be empirically proven in the case of minimally conscious patients who are unable to consistently interact with their environment. It requires that medical professionals are able to assess whether the patient disposes of the capacities to *understand, appreciate, and reason*, which is significantly more difficult to do in patients with impaired consciousness. Nonetheless, Peterson and colleagues emphasize that it is not impossible. According to them, existing assessment tools such as the MacCAT-T can still be used, if they are modified so as to align with the technical and methodological limitations that come with neuroimaging communication. The general idea is that it should be possible to modify the semi-structured interview MacCAT-T in a way that accommodates patients with cognitive and communication impairments. In 2013, Peterson and colleagues suggested a modus operandi to decompose the four mental capacities required for decision-making capacity–understanding, reasoning, appreciating, and communicating–in cognitive functions underlying these capacities (2). These highly specific cognitive functions then “may be traceable through both the mental imagery paradigm and a number of alternative passive or anatomical techniques” [(2), p. 6]. For instance, the ability to understand could be decomposed–without claiming an exhaustive analysis–into the abilities to store information, to sustain attention for periods of time, and to form new memories *post ictus*. A neuroimaging-based examination then can establish whether patients suffering from PDoC dispose of these abilities and thus possess the cognitive abilities required for decision-making capacity.

In a more recent article, Peterson and colleagues suggest a different framework for aligning the specificities of the procedure of obtaining informed consent *via* neuroimaging, with the requirements for informed consent. Accompanied by a disclaimer that their “proposal is speculative, has not been applied in practice, and might ultimately be unfeasible” [(18), p. 13], the authors set forth an interpretation of the MacCAT-T tailored to neuroimaging techniques in the form of a vignette approach. In short, they suggest that it might be possible to formulate highly refined snapshots of medical information–vignettes–and then ask the patient yes-or-no questions about these vignettes. The answers to these questions will reveal information relevant to the patient's decision-making capacity as, for example, “correctly responding to factual questions might demonstrate the ability to understand relevant medical information, while correctly responding to questions about hypothetical scenarios might demonstrate consequential reasoning, or the ability to appreciate how different treatment decisions will affect one's medical condition” [(18), p. 8].

This straightforward approach seems very appealing. However, further research, predominantly in psychiatry, will be needed to develop robust criteria that can be used to assess the cognitive abilities of patients with minimal (covert) consciousness, and that can be integrated in a decision-making assessment tool such as the MacCAT-T. An additional challenge is that factors other than a patient's cognitive abilities may also impact their decision-making capacity. Factors like “delusions, hallucinations and affective disorder which may manifest in depression, manic illnesses, and lack of maturity, as well as […] emotional and cognitive maturity” [(32), p. 230] may impact decision-making capacity, but are rather difficult to assess in a patient suffering from PDoC. It is important to emphasize that the mere observation that someone suffers from a psychiatric disorder, does not allow for definite conclusions on their decision-making capacity. For instance, most patients suffering from major depression reportedly have the required decision-making capacity to be considered competent (33). However, when treating a patient with impaired consciousness who has a history of psychiatric pathology, it may be appropriate to conduct a mental health screening before subjecting them to a more general competence assessment. Especially when competence is examined in view of decisions with a major impact–such as end-of-life decisions–a preliminary mental health assessment is advisable. This would require integrating a mental health assessment within the neuroimaging paradigm. To that aim, mental health assessment tools such as SCL-90 (Symptom Checklist 90) or BDI-21 (Beck Depression Inventory, more specifically targeting depression) should be reshaped, so that they can be used by relying on yes-or-no-questions.^10^ Furthermore, for patients who do not have a history of psychiatric disorder, it could be argued that a psychiatric disorder that impacts their decision-making capacity would already be revealed in a general competence assessment using the MacCAT-T. For instance, in patients suffering from major depression, it is observed that their capacity to appreciate information might be impaired (34). When this would be the case, this may be detected during a general competence assessment.

In addition to the conceptual and methodological issues that come with the translation of the existing competence-assessment framework into a framework tailored to neuroimaging communication, there are other practical and ethical issues that should be kept in mind. For instance, an important challenge is that communication *via* neuroimaging can currently only proceed on the basis of yes-or-no questions. This leaves little room for nuance and provides medical professionals with very little input to thoroughly judge the patient's cognitive abilities. Moreover, currently, communication *via* neuroimaging methods takes a very long time while only allowing for a limited number of questions (18). Furthermore, communication will always be entirely determined by the questions the medical professional chooses to submit to the patient (19). This puts an enormous responsibility on the medical professionals as they have to make sure that all concerns their patient might have are addressed in a way that is clear for the patient, as the latter cannot ask for any clarification. In addition, potential biases on the part of the medical professionals may cause concerns that may be important to the patients not to be included in the questions. These issues form important limitations as sufficient time and nuance, and the possibility for the patient to actively participate, are essential to assess decision-making capacity. Another related concern is the risk of mis- and overinterpretation. Flawed interpretation of neuroimaging output may lead to false negatives or false positives in the context of yes-or-no communication. Since medical professionals can only rely on brain recordings, there is the real risk that they read too much in the brain patterns (35). Mis- or overinterpretations, when involving major medical decisions, might result in a considerable infringement on a patient's autonomy, physical integrity, and well-being.

In addition to the limitations inherent to communication *via* neuroimaging, there are other ethical issues to consider. Firstly, there is an obvious paradox in that neuroimaging itself constitutes a medical treatment for which informed consent is required. Therefore, medical professionals should in fact obtain informed consent before using neuroimaging to obtain informed consent for another medical treatment. As Stout observes, “if, in a given case, NTA [Neurotechnological Thought Apprehension] is the only means of assessing capacity, and using it requires decisional capacity, then we are left with a paradox” [(36), p. 29]. This argument might render any capacity assessment *via* neuroimaging unethical. Undergoing neuroimaging, however, constitutes a rather non-invasive procedure where the risk of physical harm is limited. Relying on substitute decision-making may thus not be problematic. In this regard, it should be pointed out that in taking this decision substitute decision-makers cannot be guided by the patient's previously expressed wishes as these neuroimaging techniques are novel and the patient's preferences in this regard will probably not be known. Consequently, they need to assess whether communicating through neuroimaging would be in the patient's best interests, which may not always be straightforward to determine.

Secondly, subjecting patients to neuroimaging techniques that *read their mind* might be a source of distress to them. For example, the fact that the patient might not be fully able to express what they actually want to express, and can only express what the questions the medical professional poses allow them to, might be a source of major frustration. In addition, knowing that they might be subjected to neuroimaging at any time, may cause discomfort and feel like a threat to their privacy. Patients with minimal (covert) consciousness are completely at the mercy of their environment–caretakers, family, physicians–for engaging in a “conversation.” Patients who might just “want to be let alone” cannot meaningfully demarcate their personal privacy sphere. Moreover, it is readily conceivable that–apart from the context of obtaining informed consent–the patient's family and close friends want to communicate with the patient, whenever possible and about less important subjects. This may put the privacy interests as well as the well-being of the patient at risk.

Thirdly, the decoding of neuroimaging output to *read peoples' thoughts* inevitably implies the collection, processing, and storage of brain data and mental data. These categories of data are very sensitive data as they may concern the core of a person's identity. A data breach could generate a severe violation of the patient's privacy and dignity. Specifically in the light of the rapid developments in neuroscience and -technology, it is difficult to adequately consider potential unanticipated uses of brain data (37). For instance, brain data obtained today in the context of obtaining informed consent, could in the future turn out to bear information on a mental disorder of the patient or on their sexual or political orientation when subjected to more advanced decoding methods. Strong data protection measures need to be in place so that this information cannot be processed by third parties against the patient's will. Although at the level of the European Union the General Data Protection Regulation (38), for instance, establishes a strong data protection framework, the specific characteristics of brain and mental data make some experts suggest that the existing data protection framework might have to be re-evaluated (39, 40).

Fourthly, another concern that was pointed out in an interview of ethicists, legal professionals, researchers, and advocacy leaders, carried out by Byram and colleagues, relates to the burden on the environment of the patient (e.g., family and friends). Where one interviewee pointed out that “bringing in that extra dimension to the decision-making would likely in some circumstances relieve some of that emotional burden I think from the loved ones” [(19), p. 617], another considered that “some families will be relieved if there are signs of consciousness, others will be horrified […] they'll still be left with difficult questions about quality of life and whether life support should be removed” (idem, p. 617). Important in this regard is the management of expectations with all parties involved in the medical decision-making process (i.e., the patients themselves, their family and close friends, and medical professionals) (41). Education and support are of key importance in this regard. As observed in the treatment of locked-in patients, uncertainty about the new situation these patients find themselves in often results in considerable distress as physicians and family members are not always well-prepared to put the patient's interests first when taking important–and emotionally loaded–decisions (42). The role of every actor involved should be clearly delineated, as the patients' proxies might remain important actors within the decision-making process. For some patients, with whom neurotechnology-enabled communication proves to be impossible, medical professionals will still have to resort to substitute decision-making. In the same way, for patients with whom such a communication is possible, there may be some situations that might call for the continuing involvement of legal representatives.

This ties in with a last important consideration, namely that of the implications of the gliding scale approach to the threshold for decision-making capacity. Less impactful decisions (e.g., the administration of pain-relieving drugs) come with a lower threshold for decision-making capacity, whereas high-stake decisions imply a higher capacity threshold. In this light, it can convincingly be argued that less impactful decisions might be eligible to be addressed by neuroimaging procedures whereas currently, “clinically relevant decisions with high-stakes outcomes (e.g., invasive procedures and end-of-life-decisions) should not be addressed through BCI neuro-imaging paradigms since the conceptual and empirical foundations of this process are not yet satisfactory established” [(2), p. 10]. Only when those foundations would be strengthened in the future, neuroimaging could be deployed in the decision-making process for more impactful medical decisions. Another important issue in this regard that cannot be left unaddressed, is the issue of end-of life-decisions. The case of vegetative patients often features as a battlefield where pro-life advocates and proponents of the right to die in dignity meet each other. Can we imagine a point where end-of-life-decisions might be left for a patient suffering from PDoC to answer solely by means of neuroimaging tools? Answering this question would require too much speculation as the full potential of neurotechnology and decoding methods is not yet known so that it is unclear how reliable answers of a patient might one day be. In any case, end-of-life-decisions are at the very end of the scale of complexity and impact of medical decisions. Consequently, this is a kind of decision for which substitute decision-making will remain an important model to obtain informed consent.

Although less impactful decisions could be taken *via* neuroimaging procedures, practical limitations may stand in the way. For instance, questions such as “Are you in a comfortable position?” or “Do you experience any pain for which you would like pain medication?” probe for experiences in the “here and now.” As long as the use of neurotechnological support tools requires as much planning as it does today, their usefulness may be limited. Nevertheless, when these tools would become easier-to-use and would require less practical management–which would be the case when, for instance, EEG-based BCI would be operationalised–this challenge may be overcome.

## Supported decision-making

Although the articulation of preferences and choices of patients with cognitive disabilities and communication impairments is rarely straightforward, adequate support may enable them to make medical decisions or, at least, allow their voices to be heard as one factor in the decision-making process. Therefore, supported decision-making could be a valuable model for some patients who are, as a result of their disability, assumed to lack the ability to make their own decisions. Hence, unsurprisingly, several scholars connect the model of supported decision-making with the use of neuroimaging in minimally conscious patients (18, 19).

Supported decision-making can be defined as “a process by which an individual who might otherwise be unable to make his or her own decisions becomes empowered to do so through support from others” [(43), p. 314]. This definition focusses on assistance or support in the most classical sense, i.e., support by one or more trusted persons in the decision-making process. These persons may assist patients by, for instance, explaining information or treatment options to the patients, interpreting their verbal or behavioral cues in order to ascertain their preferences, and communicating their decisions (43). However, support is a broad notion that can also refer to support mechanisms other than human assistance. Support can also consist in “non-conventional methods of communication, especially for those who use non-verbal forms of communication to express their will and preferences” [(44), p. 13]. This broad interpretation paves the way for a wide range of supported decision-making procedures. In the paper by Peterson and colleagues on the one hand, and Byram and colleagues on the other hand, the way in which the concept of “support” is analyzed in relation to neuroimaging procedures differs. On their part, Byram and colleagues suggest that medical professionals using neuroimaging technologies and decoding methods to communicate with patients with minimal (covert) consciousness may in themselves be considered a form of support. By contrast, Peterson and colleagues elaborate on supported decision-making where the support consists of family or trusted friends acting as a means to somewhat counterbalance the existing concerns surrounding communication *via* neuroimaging. These persons can potentially function as a kind of safeguard as they can indicate whether the answers obtained *via* neuroimaging are in line with the preferences and beliefs held by the patient, and they can inform the medical professionals so that these can ask more specific follow-up questions in order to ensure that the answers provided are reliable and correspond to the patient's values (18).

Acknowledging that as a decision-making method supported decision-making might not be a brand-new concept–as patients rarely make decisions without consulting their family or friends –, it has as a legal notion swiftly occupied a central place within disability law since the introduction of the Convention on the Rights of Persons with Disabilities (CRPD). The basic principles articulated in the CRPD–dignity, autonomy, freedom to make one's own choices, and equality^11^-, are important in contexts where substitute decision-making is a default regime for persons lacking decision-making capacity. Article 5(3) CRPD, for example, holds the obligation of reasonable accommodation in order to ensure equal treatment of persons with a disability. This also relates to possible accommodations to enhance their decision-making capacities and support them in providing informed consent.

In this regard, Article 12 CRPD is especially important. Article 12(2) CRPD states that *States Parties shall recognize that persons with disabilities enjoy legal capacity on an equal basis with others in all aspects of life*. This provision generated a real paradigm shift as it resulted in a central role for supported decision-making within international disability law as a tool to assure equal rights to make decisions (45). At the same time, it created a general skeptical attitude toward substitute decision-making. The profoundness of this shift is apparent in that article 12(2) CRPD “turned the practice of supported decision-making into a human rights imperative” [(46), p. 1]. The UN Committee on the Rights of Persons with Disabilities (47), followed by the Special Rapporteur on the Rights of Persons with Disabilities (48), and the UN High Commissioner for Human Rights (49), go even as far as stating that Article 12 CRPD implies the total rejection of the implementation of any legal framework for substitute decision-making on behalf of persons with disabilities. In their view, a competence model is undesirable as no one can possibly lose their legal capacity because of a deteriorated decision-making capacity (25). However, such an absolute abolition of substitute decision-making is, rightfully, met with considerable criticism (50, 51). A total rejection of substitute decision-making cannot be supported as it is simply unfeasible and, in addition, not in the best interests of people with a disability. While a default regime of substitute decision-making might be an unreasonable restriction of the autonomy of the patient, eliminating the possibility of substitute decision-making altogether does not strike a fair balance either as it might be detrimental to the well-being of the patient (52). The radical position of rejecting any form of substitute decision-making cannot be endorsed since for those patients who clearly and persistently lack decision-making capacity, substitute decision-making may be the only available option.

Nonetheless, substitute decision-making should only be considered as an option of last resort for those patients for whom support within their decision-making process does not result in a meaningful and reliable expression of their will. This more conservative approach to Article 12 CRPD still allows for the promotion of supported decision-making as the default decision-making procedure, to be pursued whenever this proves to be possible. This procedure then does not start by examining the patient's capacity (53), but rather by reflecting on the question what means would be needed to optimally strengthen the psychological abilities and communication skills of the patient with impaired cognition to exercise their legal rights to their fullest. Article 12 CRPD then implies that State Parties must actively provide necessary support to patients who need assistance in order to fully enjoy their autonomy and to exercise their legal capacity in an equal way as non-disabled persons (54). It requires States “to take care, to ensure all citizens are considered when developing legislation, policy and practice guidelines around supported decision-making. This includes those who historically, have not been invited to the self-determination ‘party' ” [(46), p. 14]. Hence, legal frameworks that currently regulate all forms of substitute decision-making in one, uniform way should be replaced–or more accurately: reconsidered–in the light of supported decision-making (47, 55).

What does this model have to offer for patients suffering from PDoC? In my view, it could have a considerable role to play since both approaches mentioned above–neurotechnologies as support, as well as support by trusted persons–are valuable and ought to be combined when first implementing neuroimaging in informed consent procedures for minimally conscious patients. The use of neuroimaging could constitute a tool which has to be used to obtain informed consent where possible. Considering the current state-of-the-art, only a small percentage of patients who are minimally conscious would benefit from the use of neuroimaging for informed consent. Nevertheless, for these patients this might be an enormous opportunity to regain a sense of control within their medical treatment trajectory, which would contribute to their physical and mental well-being (43). Therefore, in agreement with Byram and colleagues, it should be stressed that “Article 12 […] could require signatories to the CRPD to provide neuroimaging as a means for PDoC patients to exercise their right to legal capacity” [(19), p. 614]. However, this technology is very novel and innovative. This results in significant risks, such as reading too much into answers obtained *via* neuroimaging or not grasping all the necessary nuances of the patients' preferences due to the basal, unilateral form of communication. Therefore, including persons close to the patients in their medical decision-making process might be necessary to guarantee an optimal reliability and consistency of the output generated *via* neuroimaging, and to counterbalance some of the moral issues that arise with this new technology.

## Conclusion

With this analysis, I aim to emphasize the importance of looking for ways to optimally empower the voices of minimally conscious patients. The fundamental principles within healthcare, bioethics, and disability law undisputedly require us to explore these possibilities to the maximum. A crucial first step in this process is promoting neuroscientific and -technological research that generates new and important insights into brain functioning and the mental capacities of patients suffering from PDoC. As described in this article, this neurotechnological research is necessary in order to develop avenues to communicate in a reliable and meaningful way with these patients. Notwithstanding major accomplishments in this regard, stable and reliable BCI-communication with patients with impaired consciousness currently remains a distant goal. This, however, should not refrain us from proactively engaging in research in psychiatry and bioethics that enables aligning the communication *via* neuroimaging and BCI technology with the requirements for valid informed consent. Furthermore, although some important steps have already been taken and neurotechnological developments look very promising, thorough ethical and legal reflection remains crucial before incorporating neuroscientific findings into regulatory frameworks regarding informed consent. This reflection is of essential importance to ensure the responsible development and implementation of procedures to include patients in their own medical decision-making so as to optimally protect their interests and strike a fair balance between their fundamental right to autonomy and their well-being.

## Author contributions

The author confirms being the sole contributor of this work and has approved it for publication.

## Funding

This research was supported by the Fund for Scientific Research of Flanders (FWO).

## Conflict of interest

The author declares that the research was conducted in the absence of any commercial or financial relationships that could be construed as a potential conflict of interest.

## Publisher's note

All claims expressed in this article are solely those of the authors and do not necessarily represent those of their affiliated organizations, or those of the publisher, the editors and the reviewers. Any product that may be evaluated in this article, or claim that may be made by its manufacturer, is not guaranteed or endorsed by the publisher.

## Footnotes

^1^I will focus on the group of patients who have been suffering from DoC for at least 28 days, since the observations and claims made in this article may be far less relevant for patients who suffer from DoC within shorter timeframes. For these patients, other issues such as the optimalisation of the recovery trajectory to regain consciousness should be the central focus, and not the development of support tools to obtain informed consent (for which time is needed to personalise these tools to the specific profile of the patient concerned).

^2^As disorders of consciousness are sitting on a gliding scale, more fine-grained classifications are made (4).

^3^This research relied on the Coma Recovery Scale Revised (CRS-R). This is a well-established standardised behavioural assessment tool that determines the patients' level of consciousness. The researchers compared clinical consensus diagnoses generated by medical professionals on the basis of bedside observations, to diagnoses derived from CRS-R assessments in order to establish a misdiagnosis rate.

^4^Also referred to as “Cognitive Motor Dissociation” (11) or “non-behavioural MCS (MCS^*^)” (15).

^5^For instance, in the study conducted by Monti et al., of the 54 patients they tested, 5 patients were able to perform the mental tasks. Of this group, 1 patient was able to answer yes-or-no questions (9).

^6^Brain Computer Interface (BCI) refers to technology that detects brain-activity signals via neuroimaging techniques (e.g., EEG), translates these signals into technical control commands, and transfers them to an external device such as a computer. BCI can be used to enable communication by recording brain-activity patterns and translating those into ‘speech' that can be transferred to an external electronic device.

^7^For example, already today, mindBEAGLE is such an EEG-based BCI that is available on the market. This neurodevice aims at assessing the level of consciousness in a patient suffering from DoC (so that an accurate diagnosis can be made), and also at engaging in communication with these patients. This commercially available device is, for instance, used in research conducted by Annen et al. (17).

^8^E.g., Article 5 Convention on Human Rights and Biomedicine (Oviedo Convention), Article 6(1) Universal Declaration on Bioethics and Human Rights, Articles 3 and 8 European Convention on Human Rights.

^9^Notwithstanding the fact that the assessment criteria might differ between different assessment models.

^10^Research into the criteria for the assessment of cognitive abilities and mental health status may benefit from including locked-in patients, as their cognitive functions are not – or far less severely – impaired, and medical professionals also rely on yes-or-no questions to communicate with them.

^11^Articulated in Article 3 of the convention on the rights of persons with disabilities.
